# Mitochondrial DNA Damage via Augmented Oxidative Stress Regulates Endoplasmic Reticulum Stress and Autophagy: Crosstalk, Links and Signaling

**DOI:** 10.1371/journal.pone.0083349

**Published:** 2013-12-13

**Authors:** Larysa V. Yuzefovych, Susan P. LeDoux, Glenn L. Wilson, Lyudmila I. Rachek

**Affiliations:** Department of Cell Biology and Neuroscience, College of Medicine, University of South Alabama, Mobile, Alabama, United States of America; University of Texas Health Science Center at San Antonio, United States of America

## Abstract

Saturated free fatty acids (FFAs) have been implicated in the increase of oxidative stress, mitochondrial dysfunction, endoplasmic reticulum (ER) stress, autophagy, and insulin resistance (IR) observed in skeletal muscle. Previously, we have shown that palmitate-induced mitochondrial DNA (mtDNA) damage triggers mitochondrial dysfunction, mitochondrial reactive oxygen species (mtROS) production, apoptosis and IR in L6 myotubes. The present study showed that mitochondrial overexpression of human 8-oxoguanine DNA glycosylase/AP lyase (hOGG1) decreased palmitate-induced carbonylation of proteins in mitochondria. Additionally, we found that protection of mtDNA from palmitate-induced damage significantly diminished markers of both ER stress and autophagy in L6 myotubes. Moreover, we observed that the addition of ROS scavenger, N-acetylcystein (NAC), to palmitate diminished both ER stress and autophagy markers mimicking the effect of mitochondrial overexpression of hOGG1. This is the first study to show that mtDNA damage is upstream of palmitate-induced ER stress and autophagy in skeletal muscle cells.

## Introduction

Increasing evidence, accumulated over the last decade, indicates that mitochondrial dysfunction and oxidative stress are important components contributing to the development of IR in skeletal muscle [1-3], but the underlying mechanisms responsible for these events are still unknown. Since IR is associated with numerous modern health problems, including type 2 diabetes and cardiovascular disease, it is an urgent priority to establish the molecular targets and upstream events that mediate the development of IR. Recently, we have reported that targeting the DNA repair protein human 8-oxoguanine (8-OxoG) glycosylase/AP lyase (hOGG1) to mitochondria protected mitochondrial DNA (mtDNA) from palmitate-induced damage, prevented mitochondrial reactive oxygen species (mtROS) generation and thus improved insulin sensitivity in L6 skeletal muscle cells [4]. Since we have recently shown that IR in skeletal muscle was associated with increased markers of both autophagy and endoplasmic reticulum (ER) stress [5], this study was designed to further evaluate the role of mtDNA damage in these processes. This study showed, for the first time, that mtDNA damage regulates ER stress and autophagy in L6 skeletal muscle cells. Since mitochondrial dysfunction [1,3], ER stress [6,7] and dysregulated autophagy [8,9] all contribute to the development of IR, this study sheds new light on the cause-effect relationships and sequence of events leading to IR, indicating that mtDNA damage is an early step in the chain of pathological events leading to IR. 

## Materials and Methods

### Materials

 Dulbecco’s modified Eagle’s medium (DMEM) was from Invitrogen (Carlsbad, CA), fetal bovine serum (FBS) was from Hyclone (Logan, UT). Palmitate, tunicamycin, bovine serum albumin (BSA) (fatty acid-free), insulin (from bovine pancreas), penicillin/streptomycin and NAC were from Sigma (St. Louis, MO). 

### Cell culture and treatment

 Rat L6 skeletal muscle cells were obtained from ATCC (Manassa, VA). Cells were grown in DMEM supplemented with 10% FBS and 50 μg/ml penicillin/streptomycin in 5% CO_2_ at 37°C. For these studies, L6 myoblasts were plated in culture dishes, 6-well or 24-well plates, and used at the myotube stage of differentiation as described previously [4,10,11]. Myogenic differentiation to myotubes was confirmed by light microscopy with morphological alignment, elongation, and fusion. A stock concentration of palmitate was prepared as discussed previously [4,10,11]. Control cells were treated with drug diluent only (2% BSA in the DMEM medium). For treatment with tunicamycin, cells were incubated with 5 μg/ml of tunicamycin for indicated time. A stock concentration of tunicamycin (Sigma) was dissolved in dimethylsulfoxide (DMSO). Control cultures, not treated with tunicamycin, received the same concentration of DMSO as in the compound treated cultures. In the Akt (Ser473) phosphorylation experiments, L6 myotubes were incubated with tunicamycin (5 μg/ml) or 1 mM palmitate for 16 h then serum starved for 2 h and incubated with 100 nM of insulin for 15 min [4].

### Adenovirus transduction of L6 myotubes

 Adenoviruses containing MTS-hOGG1 and GFP were kindly provided by Dr. Mark Kelley (Indiana University) and all transductions and virus-containing culture techniques were done as described previously [4]. 

### Subcellular Fractionation, Protein isolation, Western Blot Analysis, and Protein carbonylation

 Mitochondrial protein fractions were isolated from one 100 mm dish of each cell type (MTS-hOGG1 transduced and GFP-only transduced cells) by differential centrifugation as described previously [4]. Total protein isolation and Western blot analysis were performed as previously described [4]. Oxidative protein carbonylation (PCO) in mitochondrial fractions were performed after Western blot by using OxyBlot Protein Detection Kit according to the manufacturer’s instructions (Millipore) as described previously [5]. The antibodies used were to actin (Sigma); phospho-Akt (Ser 473), total Akt, pPERK, PERK, pEIF2α, EIF2α, pFox3Oa, Fox3Oa, Bnip3, ubiquitin (Cell Signaling), lamin A (Santa Cruz Biotechnology), Cox IV, an Subunit IV (Mitosciences); LC3 (Novus Biologicals); PINK1 (Abcam). Complexes formed were detected with horseradish peroxidase conjugated anti-mouse IgG or anti-rabbit IgG antibodies (Promega, Madison, WI) using chemiluminescent reagents (SuperSignal, Pierce, Rockford, IL). Where indicated, the resultant band images were scanned and analyzed using Fujifilm Image Gauge Version 2.2 software. For all densitometry analyses of Western blots, values are presented as fold induction over corresponding control data normalized to the actin level. 

### Statistical analysis

 Data are expressed as means ± SE. Differences between all groups were assessed using one way ANOVA (GraphPadPrism) followed by Bonferroni analysis where appropriate. Statistical significance was determined at the 0.05 level.

## Results

### Mitochondrial overexpression of hOGG1 protected against palmitate-induced mitochondrial protein carbonylation in L6 myotubes

**Figure 1:**
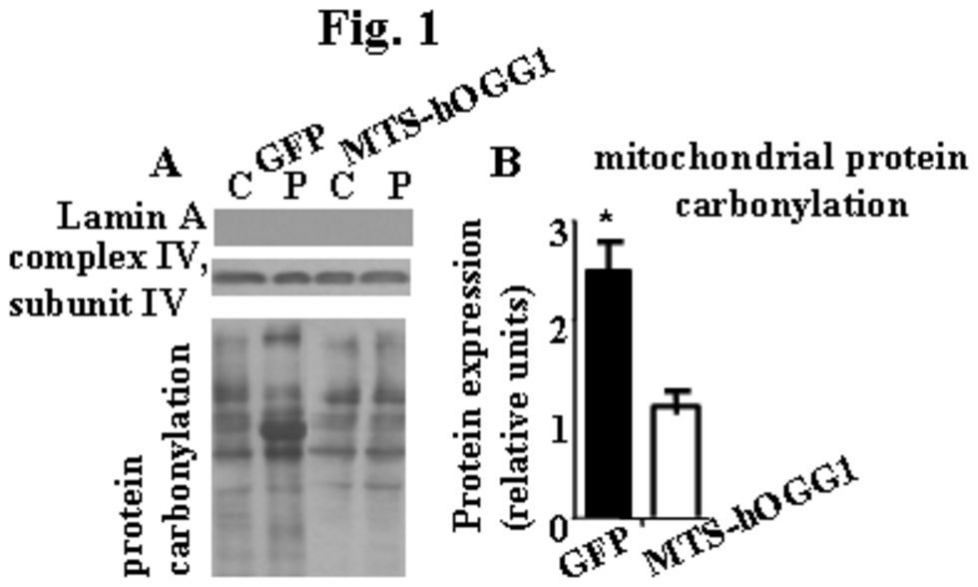
Targeting of hOGG1 to mitochondria in L6 myotubes protected against palmitate-induced mitochondrial protein carbonylation. MTS-hOGG1 or GFP transduced L6 myotubes were exposed to control medium (C, 2% BSA) or medium containing 1 mM palmitate (P). (A). Mitochondrial fractions were isolated and analyzed by Western blot with the indicated antibodies. Lamin A was used as a marker for nuclear proteins, and subunit IV of mitochondrial complex IV as used as mitochondrial marker. No nuclear contamination was detected in the skeletal muscle cell mitochondria. The values for densitometry are presented as fold induction over corresponding control data and are the means ± SE. (B). (n > 3). *p < 0.05 *vs* all other groups.

 First, to further confirm that protection against mtDNA damage prevents palmitate-induced oxidative stress, we evaluated levels of PCO using mitochondrial fractions isolated from MTS-hOGG1 and GFP transduced cultures (Figure 1A and B). As expected, PCO levels were significantly reduced in mitochondrial proteins isolated from MTS-hOGG1 as compared to GFP cultures (Figure 1A and B). 

### Targeting of hOGG1 to mitochondria of L6 myotubes protected against ER stress

**Figure 2:**
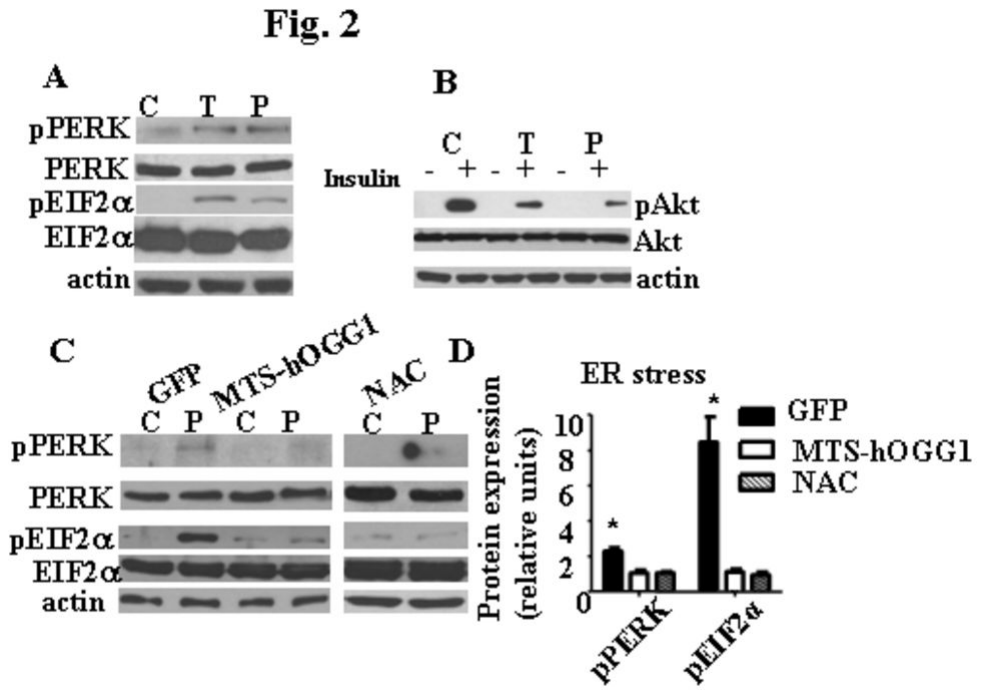
Mitochondrial hOGG1 protected against ER stress. Treatment of L6 myotubes with 1 mM of palmitate (P) or 5 μg/ml of tunicamycin (T) for 24 h induced phosphorylation of both PERK and EIF2α (A). Treatment of L6 myotubes with tunicamycin (T) or palmitate (P) reduced insulin-stimulated phosphorylation of Akt (Ser473) (B). For both A and B, control medium (C, 2% BSA) was used. Targeting of hOGG1 to mitochondria in L6 myotubes ameliorated palmitate-mediated activation of ER stress markers. MTS-hOGG1 or GFP transduced L6 myotubes were exposed to control medium (C, 2% BSA) or medium containing 1 mM palmitate (P) for 24 h (C and D). Total cell lysates were isolated and analyzed by Western blot analysis with the indicated antibodies. Representative blots (C) and values of densitometry data (D) from at least three independent experiments are shown. The values for densitometry are presented as fold induction over corresponding control data normalized to the actin level. In addition, for phosphorylated proteins, the values from densitometry from three independent experiments for each phosphorylated protein were normalized to the level of corresponding total protein, and expressed as fold induction over corresponding control data normalized to the actin level. (n > 3). *p < 0.05 *vs* all other groups.

 Next, we sought to evaluate whether palmitate induced ER stress in skeletal muscle cells, since the activation of ER stress and its contribution to the development of IR in skeletal muscle/skeletal muscle cell has been debated, with some studies showing that palmitate [12] and high fat diet [5,7] induced ER stress in mouse skeletal muscle, whereas other failed to show activation of ER stress markers in skeletal muscle of obese mice [6], as well as of insulin resistant patients [13]. Therefore, to establish whether palmitate activates known markers of ER stress in L6 myotubes, cultures were treated with 1 mM palmitate or 5 μg/ml of tunicamycin, a potent ER stressor and also, an inducer of IR in L6 myotubes [14]. Activation of several ER stress markers was demonstrated after 24 h of palmitate treatment (Figure 2A). Additionally, we have compared how pretreatment with tunicamycin or palmitate affected insulin-stimulated phosphorylation of Akt (Ser473) (Figure 2B). As shown in Figure 2B, treatment with both palmitate and tunicamycin significantly reduced insulin-stimulated pAkt levels. Moreover, we found that protection of mtDNA from palmitate-induced damage significantly reduced markers of ER stress, such as phosphorylated PERK and EIF2α (Figure 2C and D). Also, our previous study [4] has shown that protection of mtDNA from palmitate-induced damage by overexpression of hOGG1 targeted to mitochondria significantly diminished palmitate-induced activation of JNK kinase, which is considered to be a marker of both increased oxidative and ER stress [15]. 

### Targeting of hOGG1 to mitochondria protected L6 myotubes against autophagy

**Figure 3:**
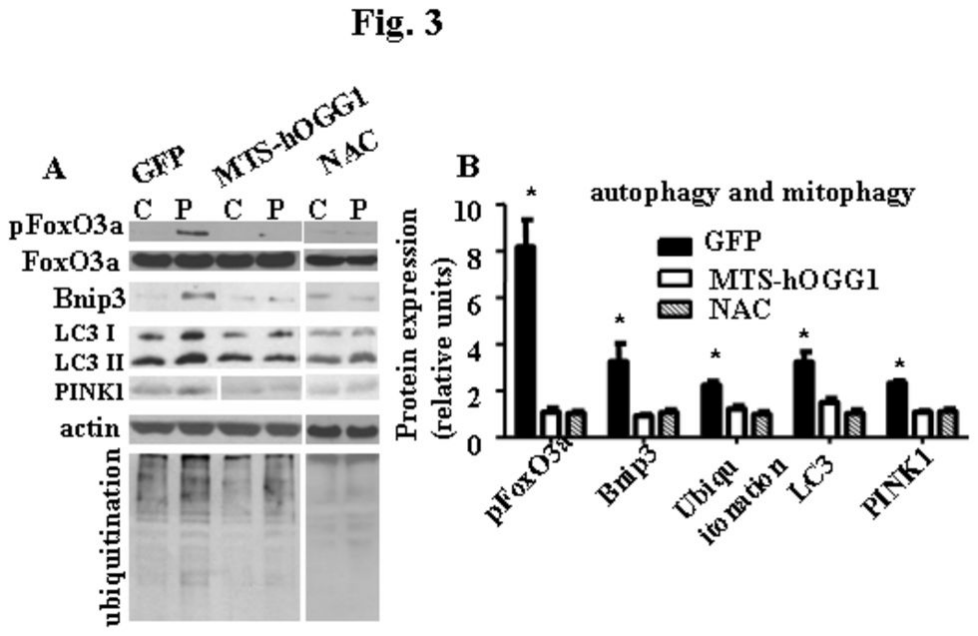
Targeting of hOGG1 to mitochondria in L6 myotubes ameliorated palmitate-mediated activation of autophagy. Representative blots (A) and values of densitometry (B) data ± SE are shown. The values for densitometry are presented as fold induction over corresponding control data normalized to the actin level. In addition, for phosphorylated proteins, the values from densitometry for each phosphorylated protein were normalized to the level of corresponding total protein, and expressed as fold induction over corresponding control data normalized to the actin level. (n > 3). *p < 0.05 *vs* all other groups.

 We found that markers of autophagy, LC3, phosphorylated FoxO3a, Bnip3, were all diminished in the MTS-hOGG1 cells as compared to GFP transduced cells (Figure 3A and B). Enhanced Bnip3 expression likely reflects the process of elimination of damaged mitochondria as a consequence of mitochondrial dysfunction and ROS production [16]. Since its expression was significantly reduced in MTS-hOGG1 cultures, it can be concluded that protection from mtDNA damage also prevented cells from mitophagy. Furthermore, we found that expression of another widely used marker of mitophagy, PINK, was also diminished in the MTS-hOGG1 cells as compared to GFP transduced cells following palmitate treatment (Figure 3A and B). Also, since we showed that the addition of ROS scavenger, NAC, to palmitate diminished both ER stress and autophagy markers, which mimicks the effect of mitochondrial overexpression of hOGG1, we therefore surmised that the protective effect of hOGG1 on the development ER stress and autophagy was at least partially through decreased oxidative stress. In addition, we have shown that MTS-hOGG1 cells have reduced level of protein ubiquitination compared to GFP transduced cells (Figure 3 A and B). Since ubiquitination of proteins is considered to be a marker of both ubiquitin-proteasome and autophagic degradation of proteins [17,18], we surmised that protection from palmitate-induced mtDNA damage in MTS-hOOG1 cells also prevented ubiquitination and, thus degradation of proteins. 

## Discussion

 Previously, we showed that protection from palmitate-induced mtDNA damage prevented mtROS generation and thus improved insulin sensitivity in skeletal muscle cells [4]. On the other hand, we recently showed that mtDNA and mitochondrial dysfunction, and oxidative stress are associated with ER stress, protein degradation/autophagy and apoptosis in high fat diet-induced IR mice [5]. Oxidative stress has been shown to be an initiator and major contributor to both ER stress [9,15] and autophagy [19], although the mechanisms that promote the activation of these signaling routes and upstream targets are not completely defined. Increased ROS are considered to act as local messengers between ER stress and mitochondria [20]. It is widely accepted that ER stress induces mitochondrial dysfunction [20,21]. Also, it has been proven that it is a two-way process [22]. Furthermore, it has been shown that mitochondrial dysfunction increased the level of ER stress markers in adipocytes [23]. The ubiquitin-proteasome and autophagy-lysosome systems are two major protein degradation pathways. During the degradation of misfolded proteins, the ER is connected to both the ubquitin-proteasome and to autophagy [21]. In addition, it has been shown that ER stress-stimulated insulin receptor degradation and consequent IR is mediated by the autophagy-dependent process [24].

**Figure 4:**
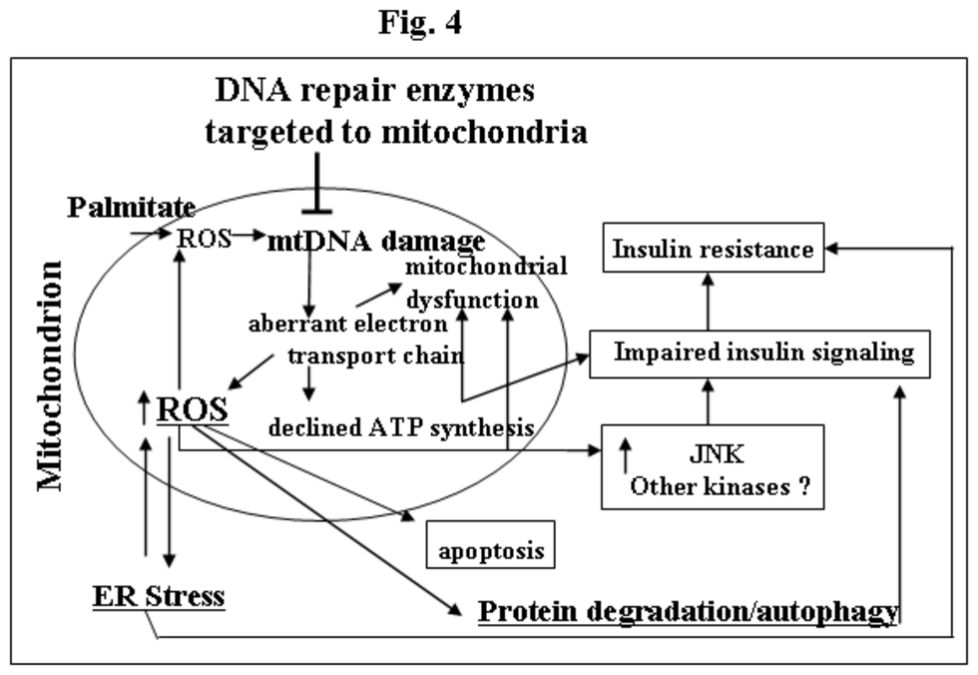
A schematic model of the proposed links between palmitate-induced mtDNA damage, mitochondrial dysfunction and ROS production, ER stress, autophagy and IR in L6 myotubes.

 Since the link between mtDNA damage and consequent mitochondrial dysfunction, and development of ER stress and autophagy has not been established yet, the current study was undertaken to examine whether protection from mtDNA damage leads to diminished ER stress and autophagy, as both processes are associated with IR in skeletal muscle *in vivo* [5]. The major principal and novel finding of this study is that palmitate-induced mtDNA damage (most likely through the generation of mtROS) is critical and proximal for the activation of both ER stress and autophagy in skeletal muscle cells. On the scheme shown in Figure 4, we presented a proposed links between palmitate-induced mtDNA, ER stress, autophagy, apoptosis and the development of IR in L6 myotubes. We suggest that palmitate-induced damage to mtDNA causes alterations in mtDNA transcription either through base mispairing, which results in defective transcripts, or decreased transcription due to polymerase blocking. Since mammalian mtDNA encodes 13 polypeptides of the electron transport chain, 2 rRNAs and 22 tRNAs, alterations in mitochondrial transcription could change electron transport complexes to cause decreased ATP production and also, lead to defective electron transfer, which would cause additional ROS production, thus establishing a vicious cycle between mtDNA damage and ROS generation, since any damage to the respiratory chain may enhance ROS production and thus heighten the oxidative stress to all other mitochondrial components, including mtDNA. This increased oxidative stress will exacerbate mitochondrial and cellular dysfunction which ultimately leads to ER stress, autophagy, and apoptosis, and to activation of stress-activated kinases which will consequently compromise insulin signaling and thus lead to IR (Figure 4). This is an additional confirmation for our long-term goal exploiting the mtDNA damage as a main object in the treatment of IR and targeting hOGG1 to mitochondria as a novel approach for treatment of obesity-induced IR and metabolic syndrome.
